# Neuromuscular block in paediatric patients undergoing airway management: a narrative review

**DOI:** 10.1097/ACO.0000000000001634

**Published:** 2026-04-27

**Authors:** Rachele Bonfiglio, Hendrik von Eichstedt, Alexander Fuchs, Nicola Disma, Thomas Riva

**Affiliations:** a Unit for research in Anaesthesia, IRCCS Istituto Giannina Gaslini, Genoa, Italy; b Department of Anaesthesiology and Pain Medicine, Inselspital, Bern University Hospital, University of Bern, Bern, Switzerland

**Keywords:** airway management, children, muscle relaxant, neonates, neuromuscular blocking agent, neuromuscular monitoring, reversal

## Abstract

**Purpose of review:**

Airway management in paediatric patients carries a high risk of respiratory complications, particularly in neonates and infants. The role of neuromuscular blocking agents (NMBAs) has evolved substantially, with increasing evidence supporting their use to optimise facemask ventilation and tracheal intubation when spontaneous breathing is not required. This review is timely as new pharmacological options and monitoring strategies have emerged to improve safety in this vulnerable population.

**Recent findings:**

Evidence from randomised trials, observational studies, and international guidelines suggests that NMBAs improve first-attempt intubation success, suppress airway reactivity, and reduce perioperative respiratory adverse events. In neonates and infants, rocuronium improves intubation conditions, and the recent approval by the Food and Drug Administration of sugammadex for use in children below two years of age expands reversal options and enhances patient safety. Despite this, residual neuromuscular block remains common, especially in the absence of objective monitoring. Quantitative neuromuscular monitoring provides superior detection of incomplete recovery compared with clinical or qualitative methods.

**Summary:**

NMBAs are a key component of paediatric airway management when spontaneous ventilation is unnecessary. Routine integration of quantitative monitoring and appropriate pharmacological reversal is essential to reduce residual block and improve patient safety.

## Graphical abstract

**Figure:**
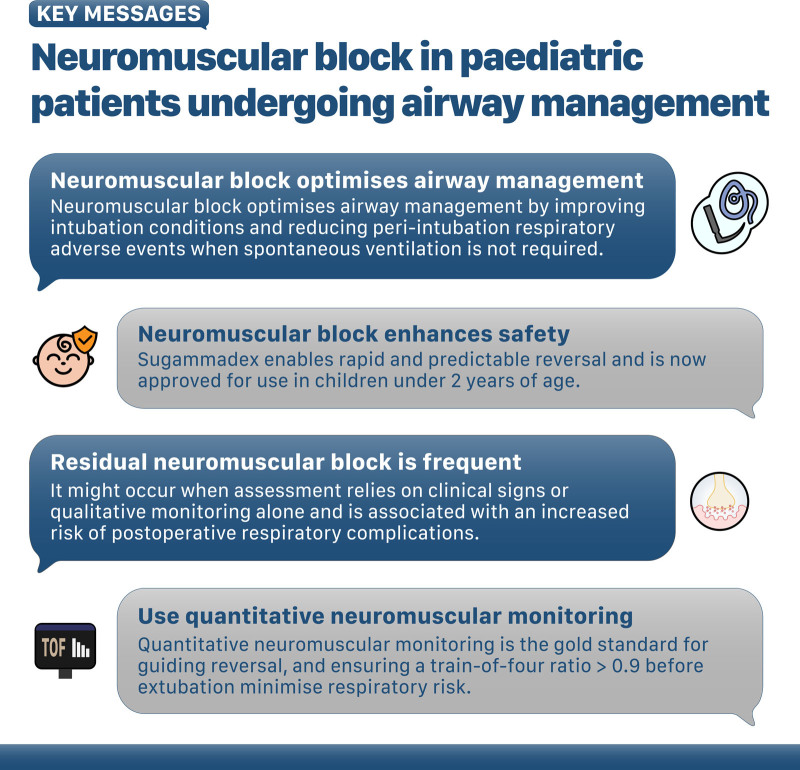

**KEY POINTS**

- Neuromuscular block improves intubation conditions in children when spontaneous ventilation is not required.
- In neonates and infants, rocuronium with available reversal with sugammadex enhance intubation success and safe extubation.
- Residual neuromuscular block remains frequent in paediatric patients, particularly when only clinical assessment or qualitative monitoring is used.
- Routine quantitative neuromuscular monitoring and patient-tailored pharmacological reversal are essential to ensure recovery and minimise postoperative respiratory complications.

## INTRODUCTION

Airway management in children remains one of the highest-risk procedures [1,2] due to their unique anatomical and physiological factors. Children have reduced functional residual capacity, high oxygen consumption [3], increased airway reactivity, and are prone to rapid oxygen desaturation.

Traditionally, when a difficult airway was anticipated, clinicians aimed to avoid a ‘cannot intubate, cannot ventilate’ situation by maintaining spontaneous ventilation to preserve airway tone and reduce the risk of upper airway collapse. However, emerging evidence from two recent systematic reviews and meta-analysis condensing the evidence from registries, observational studies, and randomised controlled trials indicates that neuromuscular block (NMB) can be advantageous when spontaneous breathing is not required [4,5]. NMB improves facemask ventilation [6], optimises tracheal intubation conditions, and reduces perioperative respiratory adverse events, informing paediatric airway management guidelines, which advocate its use [7,8].

Despite these advancements, significant variability persists in the use of NMB, neuromuscular monitoring (NMM), and reversal [9]. This is concerning, as children, particularly neonates and infants, exhibit unpredictable pharmacokinetics and pharmacodynamics, making objective monitoring essential. Residual NMB remains a preventable contributor to respiratory complications, underscoring the need for appropriate, timely reversal.

This narrative review synthesises current evidence on NMBAs during paediatric intubation and extubation, highlights age-specific considerations with emphasis neonates and infants, and outlines the critical roles of monitoring and reversal in reducing complications associated with residual paralysis.

## METHODS

The databases Ovid Medline, PubMed, Web of Science, and Embase were searched independently by RB, EvH, TR, and AF.

We conducted a comprehensive review of the literature reporting the use of NMBAs in children no more than 16 years. The following search terms were chosen: ‘neuromuscular blocking agents in children’, ‘neuromuscular drugs in paediatric anaesthesia’, ‘neuromuscular block monitoring’, ‘neuromuscular paralysis in paediatric’, ‘curarisation in paediatrics’, ‘reversal of neuromuscular blocking agents’, and no date limit was set for the search. The search was limited to the following criteria: age range (0–16 years), NMBA focus, study design (Randomized controlled trials, observational studies, systematic reviews, and case reports), and availability of full text. No language restrictions were applied.

We identified and retrieved 128 articles, and their full texts were reviewed for relevance to the objectives of this review.

## PHARMACOLOGY OF NEUROMUSCULAR BLOCKING AGENTS AND REVERSAL

NMBAs interrupt neuromuscular transmission by acting on nicotinic acetylcholine receptors at the neuromuscular junction. They are broadly divided into depolarising and nondepolarising agents.

### Depolarising agents

Succinylcholine, the only depolarising NMBA, acts as an acetylcholine analogue and produces sustained depolarisation, resulting in fasciculations followed by flaccid paralysis. Its rapid onset and short duration make it attractive in emergency scenarios, but adverse effects (i.e. hyperkalaemia, bradycardia, malignant hyperthermia susceptibility, and increased intracranial/intragastric pressure) have significantly restricted the use in children [10].

### Nondepolarizing agents

These are competitive antagonists of acetylcholine which include: intermediate acting (Rocuronium, Vecuronium, Atracurium and Cisatracurium) and long acting (Pancuronium) drugs. Rocuronium is the most frequently used NMBA due to its reliable onset, ease of titration, and reversibility with sugammadex. Rocuronium in high dosage (1.0–1.2 mg/kg) offers fast onset (<60 s) and thus can be used for rapid sequence intubation.

### Reversal of neuromuscular block

Neuromuscular block can be pharmacological reversed through different mechanisms:

- **Acetylcholinesterase inhibitors**

Neostigmine increases acetylcholine concentration at the neuromuscular junction providing competitive reversal to NMBAs. However, its non-selective action may produce bradycardia, bronchoconstriction, or gastrointestinal symptoms and requires coadministration of an anticholinergic agent. Additionally, neostigmine is ineffective in reversing deep paralysis.

- **Sugammadex**

Sugammadex selectively encapsulates aminosteroidal NMBAs (rocuronium > vecuronium), enabling rapid and predictable reversal, even from profound block [train-of-four (TOF) 0, post-tetanic count 0]. Due to the favourable safety and efficacy, its usage in children less than 2 years of age was recently approved by the FDA [11]. Sugammadex is associated with a lower incidence of PONV and a significantly shorter period of reversal from NMB in comparison to neostigmine in adult and paediatric patients [12]. Sugammadex is generally well tolerated, but reported complications and adverse events include hypersensitivity, bradycardia, transient hypotension or rare anaphylaxis (estimated incidence ranging from 0.014% to 0.0014%) [13].

In some cases, after administration of sugammadex, a rebound effect of the NMB has been reported [14]. However, this appears mainly associated with high intraoperative doses of rocuronium and under-dosing of sugammadex in absence of NMM, suggesting to a residual paralysis rather than true ‘recurarization’.

### Facemask ventilation

In difficult paediatric airway management, facemask ventilation is the critical step that determines the subsequent pathway for securing the airway [15]. Isolated difficult facemask ventilation is uncommon in children with normal airways (0.6% of cases). The coexistence of difficult facemask ventilation and difficult tracheal intubation may rise to 9% in selected high-risk cohorts, such as those included in difficult airway registries [16,17], or in children with severe obesity where the prevalence of difficult facemask ventilation is around 3% [18].

There are few studies and evidence remains conflicting regarding the benefit of NMBAs in children difficult to ventilate. In critical ill patients, data from the National Emergency Airway Registry for Children, showed that a higher incidence of perceived difficult mask ventilation was associated with the absence of NMB [19]. In a secondary analysis from the PeDI registry [20] the administration of NMBAs improved or did not affect the ease of ventilation in 90% of children with difficult airway and worsened it in only 10%. In the 54 patients in whom mask ventilation was impossible, after NMBA (26%) the ease of ventilation improved or was not affected in 12 patients (86%) and worsened in the remaining two patients (14%). These findings suggest that administration of an NMBA may serve as an effective additional management strategy in cases of unanticipated difficult mask ventilation.

### Tracheal intubation

Attempting more than two direct laryngoscopies in paediatric difficult intubation increases the risk of failure and severe complications [21]. Therefore, optimising intubation conditions to maximise the probability of first-attempt success is essential to avoid oedema, trauma, secretions, and bleeding that can further worsen laryngoscopy. Recent studies show that the use of NMBAs during intubation is associated with a reduced incidence of perioperative respiratory adverse events (odds ratio = 0.6 [95% confidence interval (CI): 0.45–0.95]) [22].

Several high-quality meta-analyses confirm the beneficial effect of NMBAs on paediatric tracheal intubation conditions [4,5]: in a systematic review and meta-analysis of children aged 0–12 years, including 1651 children, Vanlinthout *et al*. demonstrated that the use of NMBAs significantly improved overall intubation conditions, increased first-attempt success rates, and reduced the incidence of unacceptable intubation conditions compared with intubation without NMB [4]. These findings were further strengthened in a subsequent network meta-analysis and meta-regression encompassing 105 trials with 8008 paediatric participants, which showed that NMBAs consistently outperformed non-NMBA techniques by providing superior laryngoscopic view, reduced patient movement, and improved intubation quality, without a relevant increase in adverse events [5]. Furthermore, NMBA administration at a light-to-moderate depth of is associated with more stable hemodynamic during induction [4] reducing anaesthetic requirements, potentially leading to fewer anaesthetic-related side effects and faster emergence [4]. A recent Cochrane systematic review [23] including 34 randomized controlled trials with 3565 participants concluded that avoidance of NMBAs was associated with increased intubation difficulty even in children with normal airways. When intubation is expected to be difficult, ventilation strategy becomes a central factor because it can either facilitate or hinder successful laryngoscopy [6]. A retrospective analysis examined 1754 paediatric patients (<18 years) with anticipated and unanticipated difficult airway management. Among 1404 patients with anticipated difficult airway, most had difficult laryngoscopy (*n* = 1204), few had difficult facemask ventilation alone (*n* = 14), or both (*n* = 186). Ventilation strategies included spontaneous ventilation (*n* = 507), controlled ventilation with NMBA (*n* = 453), and controlled ventilation without NMBA (*n* = 329). Non-severe complications were more frequent with spontaneous ventilation (22.5%) than with controlled ventilation with (13.3%) or without NMBA (13.4%). Hypoxaemia and airway reactivity were significantly more common with spontaneous ventilation, while laryngospasm was least frequent with NMBA use. Differences in complications diminished when airway reactivity was included in the model, highlighting inadequate suppression of airway responses as a key driver of complications [6].

The Paediatric Difficult Airway Society Guidelines [24] recommend establishing an adequate NMB to maximise first-attempt success without significant physiological derangement during intubation. However, while certain situations (large head and neck masses or profuse bleeding) require maintaining airway muscular tone, expert clinical judgment remains essential in all cases of difficult mask ventilation and intubation. It is fundamental to maintaining an adequately deep plane of anaesthesia during laryngoscopy [6].

### Neonates

Neonates and infants have distinct anatomical and physiological characteristics that place them at particularly high risk for respiratory (e.g. hypoxaemia leading to bradycardia, laryngospasm, bronchospasm) and traumatic complications (e.g. oedema, bleeding) during airway management [25]. Historically, awake intubation was frequently performed in this population due to fear of adverse drug effects, and medicolegal implications associated with managing these vulnerable children. Recently, the European Society of Anaesthesia and Intensive Care (ESAIC), in collaboration with the British Journal of Anaesthesia, published specific guidelines for this group of young patients, considered the most fragile even in healthy conditions [7,8]. They recommend the use of adequate sedation or general anaesthesia during airway management (1B), and the administration of neuromuscular blockade before tracheal intubation when maintaining spontaneous breathing is not required (1C). The authors emphasise that the risks and benefits of neuromuscular blockade should be evaluated on an individual basis, considering patient factors and the clinical team’s experience. In this population, several randomised controlled trials demonstrate that rocuronium improves first-attempt intubation success, even at a dose of 0.2 mg/kg [26,27]. However, the routine use of NMBAs in settings as NICU, remains controversial and further studies are needed to clarify risks and benefits.

The recent approval by the FDA of sugammadex for infants less than 2 years of age further enhances safety. Reported cases of re-paralysis appear to be related to redistribution of unbound rocuronium into the plasma secondary to an imbalance between rocuronium dosing and sugammadex administration [28] especially when very high doses of NMBAs were administered.

### Neuromuscular blocking agents monitoring

Neuromuscular monitoring plays an important role in paediatric anaesthesia. In children, especially neonates and infants, the onset and recovery profiles of NMBAs are less predictable, increasing the risk of residual NMB [29,30] and postoperative pulmonary complications [31].

Two forms of NMM are used in clinical practice: qualitative and quantitative. While qualitative NMM relies on a peripheral nerve stimulator with visual or tactile evaluation of muscle twitches, quantitative monitoring uses acceleromyography (AMG), mechanomyography, or electromyography (EMG) to provide objective numerical assessments. Qualitative monitoring depends on the provider’s subjective interpretation of the resulting muscle response, which is often inaccurate [31,32]. In contrast, quantitative monitoring, particularly EMG-based devices, has demonstrated high reliability, with greater than 90% success in children greater than or equal to 20 kg [33], and high feasibility even in infants less than 10 kg [34]. EMG-based NMM deliver small electrical nerve stimulations and measure the muscle’s resulting electrical activity. In a TOF test, four rapid stimuli produce diminishing muscle responses as blockade increases. The TOF ratio – twitch 4 divided by twitch 1 – indicates recovery, with values greater than or equal to 0.9 generally signifying adequate return of neuromuscular function.

Among the screened literature, only 7 studies [29,31–36] explicitly reported the use of NMM in 497 paediatric patients aged 25 weeks of gestational age to 17 years (Table 1). Six of these studies [29,31–35] concluded that clinical signs and qualitative monitoring are insufficient for reliably identifying residual NMB. This inadequacy stems from several factors. First, subjective visual assessment frequently fails to detect TOF fade when TOF ratio is greater than 0.4 [33], and significant weakness may remain unnoticed. Second, clinical assessment demonstrates low sensitivity, ranging from 10% to 30% [33], making it unreliable as an indicator of neuromuscular recovery. Third, qualitative nerve-stimulator responses rely on subjective interpretation, leading to a large proportion of unrecognised residual NMB [32]. All reviewed studies highlight that quantitative neuromuscular monitoring in small children is feasible and more accurate.

**Table 1.** Summary of studies reporting on neuromuscular monitoring in paediatric patients

Study	Design	Population	Setting	Intervention	Comparator	Monitoring	Primary outcome	Results
Soffer *et al*.[29]	Prospective observational, open-label pilot study	19 infants, 25 weeks GA–<6 months	NICU/OR	NMBA exposure group: Rocuronium bolus	NMBA exposure group: Vecuronium infusion	TOF tri-axial AMG (StimPod NMS 450, Xavant Technologies, South Africa) + visual thumb twitch (al ulnar nerve stimulation, adductor pollicis muscle response)	Time to recovery TOF ratio >70%	Prolonged recovery: 14 h (roc) and 34 h (vec)
Unterbuchner *et al*.[31]	Prospective observational study	89 children 4 months–17 years, ASA I–III	OR/PACU	Routine NMM (quantitative, qualitative or none)	No control group	AMG TOF-Watch SX (Organon Ltd., Dublin, Ireland), TOF count, TOF ratio and post‑tetanic count measured at adductor pollicis muscle stimulating ulnar nerve	RNMB incidence; monitoring rates	Incidence of RNMB 10.1% (*n* = 9) (among RNMB patients, 22.2% had qualitative NMM (*n* = 2), 44.4% had quantitative NMM (*n* = 4), and 33.3% had no NMM (*n* = 3)); monitoring rates: quantitative: 65.2%, qualitative: 5.6%
Faulk *et al*.[32]	Prospective observational, pragmatic cohort study	74 children, 2–17 years, ASA I–III	OR/PACU	Routine clinical antagonism using sugammadex (mean dose 2.5 mg/kg, IQR 2.0–4.0)	Routine clinical antagonism using neostigmine (mean dose 0.05 mg/kg, IQR 0.04–0.07)	Qualitative monitoring (nerve stimulation/clinical signs) during emergence + TOF EMG in PACU (TwitchView, Blink Device Company, Seattle, WA; TetraGraph, Senzime AB, Uppsala, Sweden)	RNMB incidence	29.7% RNMB with neostigmine; 0% with sugammadex (increasing weight associated with higher RNMB incidence)
Owusu‑Bediako *et al*.[33]	Prospective observational study	100 children, 4–17 years	OR	New EMG-based TOF monitor (TetraGraph)	Qualitative TOF monitoring (peripheral nerve stimulation) or other quantitative monitoring system (e.g. AMG)	EMG TOF (TetraGraph, Senzime BV, Uppsala, Sweden) with adult electrode array (TetraSens) + PNS (ulnar nerve recording abductor digiti minimi)	Feasibility of EMG monitoring, recovery rate TOF ratio ≥0.9	93–95% successful recording with EMG; mean TOF ratio 90.1% at extubation
Kalsotra *et al*.[34]	Prospective observational study	51 children, 0.2–7.9 years	OR	Novel EMG monitor and newly paediatric-sized sensor	Adult-sized EMG sensors and comparison of two subgroups: ≤15 vs. >15 kg	EMG TOF with paediatric sensor (TetraGraph EMG Monitor with TetraSens electrodes)	Feasibility and reliability of new EMG monitor and paediatric sensors in small children	Monitoring feasible down to 4.2 kg; TOF ratio near 100% and recovery ~98%; no technical complications occurred
Tobias *et al*.[35]	Prospective, comparative analysis	50 children, mean 3 years, ASA I–III	OR	Hand vs. foot quantitative EMG monitoring simultaneously in same patients	No control group	EMG monitoring (TetraGraph monitor system Senzime AB) at adductor pollicis muscle and at the flexor hallucis brevis (with TetraSens Paediatric Senzime AB) sensors	Onset/recovery timing, baseline T1 amplitude	Foot recovery delayed by 191 s; T1 incomplete
Park *et al*.[36]	Prospective, randomised, double-blind trial	114 children, 1–9 years, ASA I–II	Ambulatory surgery	Group I: Rocuronium 0.3 mg/kg i.v. (during sevoflurane induction)	Group II: Alfentanil 14 µg/kg i.v.; Group III: Propofol 2 mg/kg i.v. (each during sevoflurane induction without NMBA)	Acceleromyography (TOF-Watch SX; Organon Ireland Limited, Dublin, Ireland) at the adductor pollicis muscle	Intubating conditions, emergence time	Rocuronium and alfentanil superior to propofol regarding intubation conditions and emergence time

EMG, electromyography; NICU, neonatal ICU; NMBAs, neuromuscular blocking agents; NMM, neuromuscular monitoring; OR, operating room; PACU, postanesthesia care unit; RNMB, residual neuromuscular block; TOF, train-of-four.

Only one study investigated the use of NMM during elective and urgent surgery: the overall monitoring rate was 70%: 65% of children received quantitative monitoring and 6% qualitative [31]. With quantitative monitoring, the incidence of residual NMB is significantly reduced, and related complications occur less frequently [37]. Faulk *et al*. reported a 30% incidence of residual NMB when only clinical signs or qualitative monitoring with neostigmine antagonism were employed, compared with 0% after sugammadex [32]. However, even sugammadex does not guarantee reliable recovery in the absence of objective NMM [32]: recovery between muscle groups may be asynchronous and residual NMB can persist despite apparently normal clinical or qualitative responses [35]. Several barriers hinder the implementation of NMM in children, including the lack of paediatric-specific devices, calibration challenges, unfamiliarity with newer systems such as EMG and time pressure in clinical practice [33,34]. However, quantitative monitoring remains the only reliable method for detecting even minimal degrees of NMB [32].

Thus, incorporating quantitative monitoring and appropriate pharmacological reversal is crucial in children to reliably guide safe extubation and reduce postoperative pulmonary complications [9].

## CONCLUSION

Neuromuscular block is a crucial part of pharmacological treatment in paediatric patients because it helps optimise conditions for facemask ventilation and tracheal intubation, whenever maintaining spontaneous ventilation is not necessary. Quantitative neuromuscular monitoring and appropriate pharmacological reversal are equally essential and significantly contribute to patient safety, particularly in neonates and infants.

## Acknowledgements

RB, AF, VH, ND and TR reviewed, selected the articles, and contributed to the text equally.

## Financial support and sponsorship

*None.*

### Conflicts of interest

*There are no conflicts of interest.*
